# A Timely Call to Arms: COVID-19, the Circadian Clock, and Critical Care

**DOI:** 10.1177/0748730421992587

**Published:** 2021-02-11

**Authors:** Jeffrey Haspel, Minjee Kim, Phyllis Zee, Tanja Schwarzmeier, Sara Montagnese, Satchidananda Panda, Adriana Albani, Martha Merrow

**Affiliations:** * Division of Pulmonary and Critical Care Medicine, Washington University School of Medicine, St. Louis, Missouri, USA; † Department of Neurology, Northwestern University Feinberg School of Medicine, Chicago, Illinois, USA; ‡ Institute of Medical Psychology, Faculty of Medicine, LMU Munich, Munich, Germany; § Department of Medicine, University of Padova, Padova, Italy; ‖ Salk Institute for Biological Studies, La Jolla, California, USA; ¶ Department of Medicine IV, LMU Munich, Munich, Germany

**Keywords:** circadian clock, critical care, COVID-19, SARS-CoV-2, nutrition, zeitgeber, rhythm

## Abstract

We currently find ourselves in the midst of a global coronavirus disease 2019 (COVID-19) pandemic, caused by the highly infectious novel coronavirus, severe acute respiratory syndrome coronavirus 2 (SARS-CoV-2). Here, we discuss aspects of SARS-CoV-2 biology and pathology and how these might interact with the circadian clock of the host. We further focus on the severe manifestation of the illness, leading to hospitalization in an intensive care unit. The most common severe complications of COVID-19 relate to clock-regulated human physiology. We speculate on how the pandemic might be used to gain insights on the circadian clock but, more importantly, on how knowledge of the circadian clock might be used to mitigate the disease expression and the clinical course of COVID-19.

## Introduction

Coronavirus disease 2019 (COVID-19) is a new pandemic respiratory illness with a highly variable clinical presentation, ranging from asymptomatic infection to profound respiratory and multiorgan failure (Wang et al., 2020b; Zhou et al., 2020a). With over 100 million people infected and 2 million fatalities, COVID-19 ranks as one of the most lethal infectious diseases in the year 2020 (World Health Organization, 2020). Amid enormous worldwide effort to stem this disease much about the pathophysiology of COVID-19 and its causative agent, severe acute respiratory syndrome coronavirus 2 (SARS-CoV-2; Zhu et al., 2020), remains to be clarified. Here and in the accompanying series of articles (Herz et al., 2021; Kronfeld-Schor et al., 2021; Sengupta et al., 2021), we discuss potential contributions of the field of chronobiology to the prevention, risk mitigation, prognosis, and rehabilitation of critically ill COVID-19 patients. This work is based on discussions at the European Biological Rhythms Society–convened workshop on Chronobiology and COVID-19 (*Chronobiology of COVID-19 CARE Conference*). Convened in July 2020, this virtual workshop brought together 250 researchers and clinicians worldwide. They engaged in a wide-ranging dialogue to consider how chronobiology and COVID-19 pathology might relate, what kinds of model systems are needed, and how clinical data might best be harnessed to explore such connections. Here, we provide a brief overview of COVID-19 with an emphasis on elements potentially sensitive to circadian regulation based on the existing pre-COVID literature. We conclude with a review of how critical illness and the intensive care setting influence circadian output, which is relevant to patients with severe COVID-19.

## A Brief Overview of COVID-19

The causative agent of COVID-19, SARS-CoV-2, is a novel beta-coronavirus that bears genetic and clinical resemblance to two prior endemic coronaviruses of global concern: SARS, severe acute respiratory syndrome, which appeared in 2002 and MERS, Middle East respiratory syndrome, which appeared in 2012 (Drosten et al., 2003; de Groot et al., 2013; Haagmans et al., 2014; Coronaviridae Study Group of the International Committee on Taxonomy of Viruses, 2020; Kim et al., 2020; Zhu et al., 2020). While all three coronaviruses produce a similar picture of hypoxemic respiratory failure in humans with a 20%-70% fatality rate once mechanical ventilation becomes necessary (Yam et al., 2003; Alsolamy and Arabi, 2015; Auld et al., 2020; Richardson et al., 2020), only SARS-CoV-2 achieved pandemic spread. This may be related to a greater ability of SARS-CoV-2 to productively replicate in the nasopharynx without inciting symptoms of systemic illness that would otherwise cause infected individuals to self-isolate (Wölfel et al., 2020). Epidemiologic information about the scope of asymptomatic spread remains incomplete due to the lack of comprehensive virus testing in most countries and a variable rate of false-negative tests. Among symptomatic patients, roughly 81% experience mild disease, 14% experience moderate symptoms requiring hospitalization, and 5% require hospitalization plus advanced respiratory support in intensive care units (ICUs; Huang et al., 2020; Richardson et al., 2020; Yang et al., 2020). While prior chronic lung disease represents a potent risk factor for severe COVID-19, most COVID-19 risk factors are extrapulmonary in nature and include older age, obesity, hypertension, diabetes, and cardiovascular disease (Wang et al., 2020a; Zheng et al., 2020).

SARS-CoV-2 infection begins in the upper respiratory tract with differentiated airway epithelial cells being the initial sites of infection, particularly multiciliated cells and secretory (club) cells (Hui et al., 2020). Infection proceeds distally down the conducting airways to the alveolar space leading to the infection of type I and type II pneumocytes as well as alveolar macrophages (Grant et al., 2020; Hou et al., 2020; Hui et al., 2020). While SARS-CoV-2 is cytopathic in immortalized cells, it is the host inflammatory response that is thought to be primarily responsible for acute lung injury and breakdown of alveolar architecture known as the acute respiratory distress syndrome (ARDS; Thompson et al., 2017; Bryce et al., 2020; Schaller et al., 2020). The advent of ARDS frequently requires the initiation of mechanical ventilation to maintain life, but the elevated airway pressure this intervention imposes is itself injurious and represents a second inflammatory “hit” (Slutsky and Ranieri, 2013). If the host immune system succeeds in controlling the viral infection early, ARDS lung pathology can fully resolve (Doglioni et al., 2020). However, in some individuals, ARDS lung pathology proceeds to a fibroproliferative stage rendering lungs mechanically stiff, permanently impairing ventilation, and unsustainably increasing the work of breathing (Thompson et al., 2017). Autopsy studies show that respiratory failure is usually the proximate cause of death due SARS-CoV-2, although in critically ill patients, multisystem organ dysfunction frequently occurs as well (Bryce et al., 2020; Schaller et al., 2020; Wang et al., 2020b).

Two forms of systemic pathology bear special mention in severe COVID-19. The first is macrophage hyperactivation, leading to the elaboration of high levels of circulating pro-inflammatory cytokines such as interleukin 6 (IL-6; Moore and June, 2020). The term “cytokine storm” has been applied to COVID-19; however, it is unclear whether this condition is unique to or even representative of most patients with this disease (Lukan, 2020; Mudd et al., 2020). Emerging evidence suggests that mechanically ventilated COVID-19 patients may have suppressed type I interferon responses, suggesting a more complex picture than simple hypercytokinemia (Giamarellos-Bourboulis et al., 2020; Hadjadj et al., 2020; Mudd et al., 2020). The second form of systemic pathology is hypercoagulation, resulting in microthrombus formation and, somewhat counterintuitively, bleeding (Al-Samkari et al., 2020; Bryce et al., 2020). While sharing features with the more commonly known disseminated intravascular coagulation (DIC), COVID-induced hypercoagulability exhibits unique aspects including preserved levels of circulating fibrinogen and massively increased levels of von Willebrand factor from activated or damaged endothelial cells (Ward et al., 2020; Zhang et al., 2020). The clinical ramifications are that COVID-19 patients have a dramatically increased risk of stroke, myocardial infarction, pulmonary and deep venous thromboembolism, and major hemorrhage (Helms et al., 2020b; Castro and Frishman, 2021). The mechanism underlying thrombosis in COVID-19 remains to be clarified, although defects in platelet function were recently described in patients (Iba et al., 2020; Zaid et al., 2020).

The prognosis of COVID-19 largely depends on the severity of respiratory failure. Patients with mild to moderate disease appear to clinically recover within 2 weeks, although viral RNA can be detected in many patients for longer periods (Sun et al., 2020; Wölfel et al., 2020). At present, patients who progress to mechanical ventilation spend a median of 10-17 days intubated (Almeshari et al., 2020). The long-term recovery of severely ill COVID-19 patients has yet to be charted, although reports of protracted debility in the weeks following hospital discharge are common (Lambert and Corps, 2020; Halpin et al., 2021). In general, survival from critical illnesses is followed by protracted functional and cognitive impairment that persist even a year after hospital discharge (Schelling et al., 1998; Bein et al., 2018).

## Brief Overview of SARS-CoV-2

**Figure 1.**
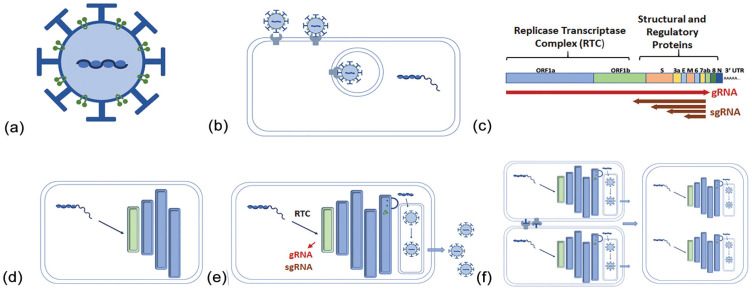
Life cycle of the SARS-CoV-2 virus. (a) Spike (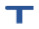), envelope, and matrix (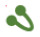) proteins are expressed on the surface of this single-stranded RNA virus. Nucleocapsid protein binds and protects the genomic RNA (gRNA) until (b) the virus enters the cells via spike protein interaction with cellular ACE2. (c) The gRNA of the positive-stranded RNA is transcribed and translated as a single ORF, yielding the RTC. Structural proteins are transcribed subsequently from the (subgenomic, sg) 5’ end of the gRNA. (d) The RTC integrates with the endoplasmic reticulum to form a double-membrane vesicle (DMV). (e) This structure produces virus which is released. (f) Local viral load leads to the formation of multinucleated giant cells through the binding of spike and ACE2 proteins on the surface of local cells. Abbreviations: SARS-CoV-2 = severe acute respiratory syndrome coronavirus 2; ORF = open reading frame; RTC = replicase-transcriptase complex; ACE2 = angiotensin-converting enzyme 2.

SARS-CoV-2 (Figure 1) is an enveloped single-stranded RNA virus that, typical of coronaviruses, is distinguished morphologically by its spike (S) protein which forms crown-like projections around the viral particles on electron micrographs (Masters and Perlman, 2013; Brahim Belhaouari et al., 2020). There are three additional structural proteins in the virion: the matrix (M) and envelope (E) proteins which reside in the membrane, and the nucleocapsid (N) protein that spools and protects the genomic RNA (Masters and Perlman, 2013; Kim et al., 2020). The S protein catalyzes viral entry and is thought to be the main determinant of tissue and species tropism (Dinnon et al., 2020; Ou et al., 2020). It is a type I transmembrane protein with a prominent extracellular region composed of two segments: an N-terminal (S1) segment that engages cell-surface angiotensin-converting enzyme 2 (ACE2), and a C-terminal S2 segment that promotes trimerization and contains a membrane fusion peptide (Cai et al., 2020; Ou et al., 2020). The bioactivity of the fusion peptide is promoted by cleavage of the S protein, which is catalyzed by extracellular proteases like TMPRSS2 or by lysosomal cathepsins (Hoffmann et al., 2020; Ou et al., 2020).

The genome of SARS-CoV-2 consists of a single positive-strand RNA roughly 30 kb in length that is dominated by a single open reading frame (Orf1a/1b) taking up two thirds of its length (Kim et al., 2020). Orf1a/1b is translated off the genomic RNA as a single polypeptide that is autoproteolyzed into multiple subunits forming the “Replicase-Transcriptase Complex” (RTC; Kim et al., 2020). The RTC is believed to be responsible for all subsequent steps of viral replication (Masters and Perlman, 2013). The structural proteins that comprise the viral capsid are expressed later in infection as a series of subgenomic RNAs (sgRNAs) that have a common 3’ end and sport an identical leader sequence derived from the 5’ end of the viral genome (Masters and Perlman, 2013; Kim et al., 2020). The leader sequence allows for capping and translation of all viral transcripts (Masters and Perlman, 2013; Kim et al., 2020).

The next step in the SARS-CoV-2 replication involves the establishment of a specialized organelle in continuity with the endoplasmic reticulum (ER) called a double-membrane vesicle (DMV) structure (Klein et al., 2020). The DMV is thought to coordinate viral replication and to sequester viral factors away from innate immune sensors in the cytoplasm (Klein et al., 2020). Of note, DMV membranes bear ultrastructural resemblance to autophagosome membranes, and autophagy markers are upregulated in SARS-CoV-2–infected cells (Stukalov et al., 2020).

Viral RNA synthesis by coronaviruses generally involves the production of a complementary negative-strand RNA template starting from the 3’ end of the viral genome (Masters and Perlman, 2013; Kim et al., 2020). Full-length templates are used for synthesis of progeny + RNA viral genomes, while the shorter sgRNA templates drive the expression of the four viral structural proteins plus accessory proteins that may play a role in virus-host interactions (Masters and Perlman, 2013; Liu et al., 2014). Rather than engaging in RNA splicing, coronaviruses have a unique method of ensuring inclusion of the genomic 5’ leader sequence in each sgRNA, which is essential for translation. Directly upstream of the body of each structural gene is a conserved “Transcription Regulation Sequence” (TRS-B), which is identical to a motif in the 5’ leader sequence (TRS-L). As the RTC engages the negative-strand RNA synthesis, the polymerase can cross over from the TRS-B motif, located upstream of the coding sequence in each nascent sgRNA template, to the TRS-L sequence, thereby appending the 5’ end of the genome to each RNA. As a result, coronaviruses rely heavily on genetic recombination to complete its life cycle and have a proclivity for rapid viral evolution (Banner and Lai, 1991). Reflecting this, the dominant SARS-CoV-2 genotype appears to have changed with enhanced infectivity as the virus spread from China to Europe and beyond (Korber et al., 2020). Even in the same infected individual, SAR-CoV-2 genomes can vary based on the anatomic location of sampling within the respiratory system (Wölfel et al., 2020).

The final step in the SARS-CoV-2 life cycle is the assembly and release of virions. For coronaviruses, structural proteins of the viral envelope (the S, M, and E proteins) are cotranslationally inserted into the ER membrane and traffic to the ER-Golgi intermediate compartment (ERGIC; Masters and Perlman, 2013; Klein et al., 2020). There, nucleocapsids consisting of progeny genomes bound by N protein interact with the envelope proteins and bud into the ERGIC lumen. Virions are believed to reach the extracellular space by bulk transport, although transport through lysosomal compartments was recently suggested as an alternative (Ghosh et al., 2020). A fraction of S protein reaches the plasma membrane, causing cell fusion with neighboring infected cells and the formation of multinucleated giant cells that are characteristic of COVID-19 lung pathology (Masters and Perlman, 2013; Bryce et al., 2020).

To summarize, SARS-CoV-2 engages infected hosts at multiple levels during severe infection, inserting itself into the host’s physiological processes, immune responses, and fundamental cellular machineries. All these layers of biological organization are influenced by circadian rhythms, whose basic structure in mammals is summarized in the next section.

### Relevant Features of Circadian Clock Organization

Circadian clocks impose a 24-h temporal structure on more processes than we are able to count (Zhang et al., 2014). In 1972, Arnold Eskin conceptualized circadian clocks as possessing an input pathway, an oscillator and output pathway (Eskin, 1979). We now know that this is an oversimplification of the system (Roenneberg and Merrow, 1998; Duguay and Cermakian, 2009; Ince et al., 2019; Reinke and Asher, 2019), but it remains a useful tool for conceptualization of circadian organization. The input pathway processes zeitgeber signals. Zeitgebers are the regular, predictable signals emanating from the environment that organisms and cells use to synchronize to time of day. For human behavior, light is the main zeitgeber, acting via specialized retinal cells that directly innervate the suprachiasmatic nuclei (SCN) of the hypothalamus (the pacemaker for human behavior). A well-known confounder of human synchronization is self-regulated and socially regulated exposure to light such that the natural photic environment is not faithfully represented to the brain. We do this by turning off the lights and closing our eyes when we sleep as well as by living indoors and therefore changing the amplitude of the light/dark cycle or, in extreme examples, by changing the timing of our light environment due to shift work. The consequences of “living at the wrong time” (shift work) are dire. Long-term shift workers have increased risks of metabolic syndrome, coronary heart disease, and certain types of cancers (Schernhammer et al., 2001, 2003, 2006; Vetter et al., 2016). The phenomenon called social jetlag refers to the generally smaller shift in timing due to the mismatch of social (e.g., school or work) and biological clocks (Roenneberg et al., 2012). Social jetlag is also characterized by behavioral and physiological deficits. It is remarkable that even relatively short bouts of desynchrony can result in metabolic disturbance. After an experimental protocol called forced desynchrony, whereby human subjects are forced into a situation where they cannot synchronize to the short or long zeitgeber cycle, individuals continue to show circa 24-h daily oscillations in physiology but prediabetic features become evident (Scheer et al., 2009). A common consequence of controlled phase shifts, as occurs in transmeridian travel or shift work, is decreased amplitude of the circadian clock (Dijk et al., 2012).

In addition to synchronization of the organism as a whole, each cell within us possesses a circadian clock. Indeed, the organismal clock is built on individual cellular circadian clocks. They respond to a cocktail of endogenous zeitgebers for their synchronization. Already in 2000, Basolobre et al. (2000b) showed that cyclic adenosine monophosphate (cAMP) analogs, dexamethasone, protein kinase C, and Ca^2+^ could synchronize and phase shift clock gene oscillations in Rat-1 fibroblasts. That this is relevant for human entrainment and clock-regulated gene expression was shown with administration of glucocorticoids once a day, in the afternoon, resulting in reentrainment of clock gene expression in peripheral blood mononuclear cells (PBMCs; Cuesta et al., 2015). Importantly, the timing of nutrient uptake has remarkable effects on the timing of clock gene expression in the liver. Experiments showed that the timing or phase of rhythmic gene expression in peripheral organs can resemble that of the SCN (entrained using light) or that of the liver (entrained using food) depending on the tissue and the relative strengths of the zeitgebers (Stokkan et al., 2001). Cellular clocks may be amplitude - attenuated by irregular zeitgeber timing (Cuesta et al., 2015), as has been observed at the organismal level, leading to a dysregulated circadian system. Obviously, entrainment or synchronization is a dominant feature of the circadian clock.

Concerning the oscillator mechanism, genetic data show transcription factor networks toggling between activation and repression to execute daily timing at the molecular level (Aryal et al., 2017). As with all other known transcription factors, the clock operates in large multimeric protein complexes. This formula creates many opportunities for fine tuning of daily timing. Changes in the timing of transcription, translation, and posttranslational modifications of any of the clock regulators, and changes in the temporal structure of zeitgebers could result in fundamental changes in characteristics of the oscillator and thus its synchronization properties.

The output pathways are like the hands of a clock, influencing clock-controlled regulation. Rhythmic gene expression—beyond the transcription factors thought to be at the center of the process—is generally tissue specific (Zhang et al., 2014). Historically, there have been reports of some tissues lacking clock-regulated gene expression, namely testis and thymus (Alvarez and Sehgal, 2005). More recently, the baboon transcriptome showed rhythms in these tissues (Mure et al., 2018). It may be that all tissues show daily temporal structures at the molecular level. By extrapolation, we suggest that all tissues will exhibit functional, tissue-specific circadian clock regulation.

### Relevance of the Circadian Clock to SARS-CoV-2 Biology and Pathology

Is there any reason to connect rhythms to the disease process—from infection to pathology—associated with critically ill COVID-19 patients? One could look at this from a level of the biology of the virus as it leads to (different degrees of) pathology to how the circadian clock may contribute to containment or exacerbation of symptoms to considerations in the clinical critical care environment. Any of these aspects may lead to novel opportunities for interventions.

Concerning viral biology, are ACE2, TMPRSS2, and FURIN clock regulated within cells? We interrogated the database that describes gene expression in 64 tissues over 24 h in young, healthy, male baboons (Table 1; Mure et al., 2018). These genes are broadly expressed, with BACE2 (the baboon ACE2 ortholog) and FURIN giving a signal in all assayed tissues. TMPRSS2 is similarly broadly expressed except in the hippocampus, the putamen, and the retina. Rhythmicity of RNA expression corresponding to these genes, however, occurs seldomly. The gene for ACE2 is rhythmic only in abdominal muscle. TMPRSS2 RNA is rhythmic only in omental fat tissue. FURIN-encoding RNA was rhythmic in nine tissues (bladder, cornea, heart, gastrocnemius muscle, testicles, thyroid, and three areas of the brain). ACE2 gene expression measured in homogenized mouse or human lung samples did not exhibit rhythmic expression (Zhang et al., 2014; Ruben et al., 2018). However, the lung has a high degree of cellular diversity, consisting of more than 40 cell types (Franks et al., 2008; Montoro et al., 2018; Reyfman et al., 2019). It remains possible that ACE2 can be rhythmically expressed in specific cells types, in patients with inhaled exposures to irritants, or in the setting of chronic lung diseases.

**Table 1.** Daily rhythms in expression of severe acute respiratory syndrome coronavirus 2–relevant genes in tissues isolated from baboons (Mure et al., 2018).

Gene	Tissue
BACE2 (BABOON ACE2)	Abdominal muscle
TMPRSS2	Omental fat
FURIN	Bladder
	Cornea
	Heart
	Medial globus pallidus
	Gastrocnemius muscle
	Prefrontal cortex
	Testicles
	Thyroid
	Ventromedial hypothalamus

Abbreviation: ACE2 = angiotensin-converting enzyme 2.

ACE2, angiotensin-converting enzyme 2, catalyzes the hydrolysis of angiotensin 2 into angiotensin 1-7, which has important anti-inflammatory properties (Kuba et al., 2013). As a result, the relationship between ACE2 levels and COVID-19 clinical severity is more complex than it appears at first glance. For example, ACE2 and angiotensin 1-7 levels tend to be reduced in older individuals, a group that it is nevertheless at high risk for clinically severe COVID (AlGhatrif et al., 2020; Miller et al., 2020). In this case, whatever benefit might be gleaned by lower ACE2 levels on viral entry is apparently outweighed by the magnification of host inflammatory responses that result from the reduced amounts of angiotensin 1-7 in older patients. While rhythmicity of ACE2 levels in the lung has not yet been shown, ACE2 shows a diurnal variation in the blood (Veglio et al., 1987). RAS has also been shown to influence the circadian clock by modulating the expression of core clock genes such as PER2 and BMAL1 (Nonaka et al., 2001).

Biological functions involved in viral processing within cells (lysosomes, endocytic trafficking, and various extracellular proteases) commonly exhibit circadian oscillations in activity (Mazzoccoli et al., 2015; Cuddapah et al., 2019; Chang et al., 2020). In general, a single rhythmic rate-limiting step in viral cell biology is sufficient to impose rhythmicity on the entire process. Therefore, there are a number of possibilities that could lead to rhythms in in vivo viral processing.

Concerning how the circadian clock may contribute to exacerbation of pathology in critically ill patients with COVID-19, several symptoms are particularly relevant. These patients may experience respiratory failure, microcoagulation events, and cytokine storm. The molecular mechanisms involved in each of these are regulated by the circadian clock. For instance, human respiratory function shows clock regulation in mechanics (airway resistance and flow characteristics), ventilatory response to hypercapnia, and airway responses to inhaled allergens (Shimoda and Semenza, 2011). Concerning hypoxemic respiratory failure that can occur in COVID-19, it is important to note that circadian clocks are sensitive to cellular oxygenation status via the action of hypoxia inducible factor 1 alpha (HIF1a; Adamovich et al., 2017; Peek et al., 2017). As such, COVID-19 respiratory failure and its treatment likely alter clock-regulated gene expression in the lung, similar to what has been described in other acute lung injuries (Jafri et al., 1992; Richardson Jones et al., 1996; Bremner et al., 2000; Numminen et al., 2000). Regarding the coagulation cascade, both clotting function and clotting factors show complex time-of-day differences. Several studies have demonstrated a diurnal rhythmicity in human platelet function (platelet counts and activation), endothelial function, and several coagulation and fibrinolytic parameters (activated factor VII, factor IX, beta-thromboglobulin, platelet factor 4, and fibrinogen; Rosing et al., 1970; Tofler et al., 1987; Akiyama et al., 1990; Willich et al., 1992, 1993; Etsuda et al., 1999; Ündar et al., 1999; Guagnano et al., 2000; Ringqvist et al., 2000; Gaenzer et al., 2001; Osmancik et al., 2004; Otto et al., 2004; Rudnicka et al., 2007). The circadian clock has been shown to modulate both intrinsic and extrinsic coagulation pathways, and the phase relations of the rhythms of different coagulation parameters may contribute to the known circadian variations in the frequency of arterial (e.g., myocardial infarction, sudden cardiac death, cerebral infarction; Willich et al., 1993; Montagnana et al., 2009) and venous (e.g., deep vein thrombosis and pulmonary embolism) thromboembolic events as well as hemorrhagic phenomena (intracerebral hemorrhage, rupture of aortic aneurysms; Maemura et al., 2006; Maas et al., 2020c).

**Figure 2.**
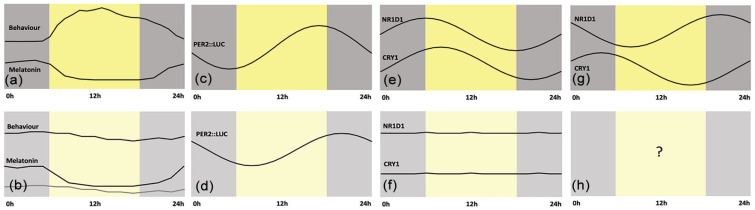
Circadian rhythms in critical illness. (a) Daily behavior and melatonin profiles in healthy individuals. (b) In intensive care unit patients with neurological and systemic critical illness, behavioral rhythms are absent and melatonin rhythms show a variety of changes depending on severity (as characterized by the Glasgow Coma Scale) and medication (the use of pressors or not). Data from Maas et al. (2020c). (c) At the level of the lung, experiments in mice show expression of the PER2::LUC transgene (d) is delayed by approximately 4.5 h in hypoxic conditions. Data from Manella et al. (2020). (e) Peripheral blood mononuclear cells show rhythms of NR1D1 and CRY1 gene expression (f) which are absent in cells from critically ill patients. Data from Maas et al. (2020a). (g) These same genes, NR1D1 and CRY1, show an 8-h difference in phase of expression in monocytes obtained from healthy human subjects. Data from Wittenbrink et al. (2018). (h) We do not know what the status of molecular rhythms is in these cells in critically ill patients. Time is expressed as external time (ExT), a convention that runs for 24 h starting at midnight (Daan et al., 2002).

With regard to the expression of cytokines, both the transcription of cytokine-encoding genes as well as stimulation of cytokine release show strong time-of-day regulation. Clock proteins are known to be activators of some cytokines and suppressors of others (Timmons et al., 2020). Clock regulation occurs in macrophages and monocytes (Figure 2) as well as for T cells, thus impacting the immune responses of these cells to pathogens (Scheiermann et al., 2018). It has been suggested that mechanically ventilated COVID-19 patients may have suppressed type I interferon responses (Hadjadj et al., 2020). It is possible that a misalignment here could influence the probability of the occurrence of a cytokine storm. This aspect is discussed in the companion paper on the clock and the immune system with respect to COVID-19.

### COVID-19 and Chronopharmacology

The development of therapies to mitigate COVID-19 is an intense area of focus. Current therapies available to critically ill patients include anti-inflammatory drugs like glucocorticoids and tocilizumab, direct antivirals like remdesivir, and SARS-CoV-2–neutralizing antibodies conferred by convalescent plasma. Whether time of day plays a role in the efficacy of these drugs in COVID-19 patients is unclear. However, the pharmacokinetics and pharmacodynamics of many drugs are known to change over the 24 h. Accordingly, so do the efficacy and tolerance to chemotherapy (Dallmann et al., 2016), and antibody response to vaccination (Long et al., 2016). A recent study has shown that approximately 50% of all currently used drugs target the product of a gene under circadian control (Anafi et al., 2017). Finally, the outcomes of certain types of surgery (e.g., aortic valve replacement; Montaigne et al., 2018) also vary across time of day. Time of day differences may reflect the time of maximum efficacy and success, or alternatively, the time of minimum adverse effects (Ballesta et al., 2017).

Recently, a prospective meta-analysis from the WHO Rapid Evidence Appraisal for COVID-19 Therapies (REACT) Working Group pooled data from seven trials totaling 1703 patients and showed that administration of systemic corticosteroids is associated with a lower 28-day mortality in critically ill COVID-19 patients (Horby et al., 2020; Sterne et al., 2020). Commonly known for their potent anti-inflammatory properties, glucocorticoids also act to synchronize the cellular circadian clock in most cells. Interestingly, dexamethasone does not act on the SCN clock, only on clocks in nonpacemaker cells (Balsalobre et al., 2000a). Theoretically, glucocorticoid administration has the potential to synchronize peripheral clocks in patients, although there are no studies demonstrating glucocorticoids can do this in the setting of critical illness. Whether the salutary effects of glucocorticoids on COVID-19 can be ascribed to circadian regulation is unknown, but it represents a topic for future investigation. Another strong synchronizer of circadian rhythms, melatonin, came to the attention of COVID-19 researchers (Cardinali et al., 2020; Zhou et al., 2020b). In a large registry of patients undergoing testing for suspected COVID-19, routine use of melatonin was statistically associated with a negative COVID-19 quantitative polymerase chain reaction (qPCR) result, suggesting melatonin might suppress SARS-CoV-2 replication (Zhou et al., 2020b). Melatonin is currently under investigation as a potential SARS-CoV-2 antiviral therapy (Rodríguez-Rubio et al., 2020).

### Implications of the Critical Care Environment and the Circadian Clock on COVID-19

The potential effect of the ICU environment on a patient’s circadian clock has been acknowledged for years. This may be especially relevant in critically ill COVID-19 patients who require a prolonged ICU stay with limited exposure to normal environmental zeitgebers. ICUs are well known to feature low, noncycling or weakly cycling light environments and excessive noise due to equipment and alarms, conditions which disturb normal sleep (Boyko et al., 2017). Furthermore, many patients are sedated due to various medical problems, interrupting behavioral control of light-dark cycles. In addition, feeding occurs intravenously and continuously, rather than by more physiological boluses. Various measures have confirmed that many ICU patients show either suppressed or inappropriately synchronized circadian rhythms. For example, Gazendem et al. (2013) showed that the phase of the rhythm in core body temperature was displaced by more hours in sicker patients, defined by a higher APACHE III score. A recent paper confirmed this general finding but used heart rate variability as a measure. Knauert et al. (2015) found that most patients (>80%) showed daily rhythms but that, already in the first 2 days in the ICU, the phase of the rhythms was misaligned relative to a control population. Based on these observations, we anticipate that many critically ill COVID-19 patients may have circadian rhythms, but the entrained phase (synchrony relative to the natural day) will not be normal and it will be difficult to predict with current information.

Certain severe illnesses may lead to disrupted circadian clock regulation independent of the environment. Patients with neurologic or systemic critical illness have been shown to rapidly enter a state of profound behavioral quiescence with the onset of illness, during which rest-activity rhythm showed severe temporal disorganization and dissociation from melatonin rhythms (Maas et al., 2020c). Dampening of melatonin amplitude has been associated with worsening encephalopathy, although improvement in encephalopathy was not associated with corresponding change in melatonin amplitude (Maas et al., 2020b). The altered oxygenation status in the lung can shift the phase of clock gene expression in the lung (Figure 2c and 2d; (Manella et al., 2020)). Furthermore, circadian gene expression becomes dampened in the ICU (Figure 2e and 2e; (Maas et al., 2020a)). A key question remains with respect to the state of the clock specifically in cells involved in acute inflammation via production of cytokines, potentially contributing to cytokine storm. Some of the highest amplitude circadian rhythms described to date occur in peritoneal macrophages (Keller et al., 2009). The monocyte subset of PBMCs show distinct phase relationships between clock gene expression relative to the whole blood cell population (Figure 2g vs 2e; (Wittenbrink et al., 2018)). It is not known how these cells function in critically ill patients or those in the ICU (Figure 2h).

Interestingly, strong physiologic stressors can induce a state of sleep-like behavioral quiescence in animal models, which occurs in tandem with protective mechanisms to restore cellular homeostasis and recover from cellular stress (Hill et al., 2014; Iannacone and Raizen, 2016; Nath et al., 2016; Trojanowski and Raizen, 2016). During critical illness, normal sleep architectures on traditional electroencephalography (EEG) frequently disappear and are replaced with various abnormal patterns that are neither sleep nor wake in a healthy state. It is therefore difficult to apply traditional EEG scoring methods for sleep and infer underlying sleep-associated neurophysiologic processes to critically ill individuals (Watson, 2007; Watson et al., 2013; Schabus et al., 2018; Wislowska et al., 2018).

Nearly 30% of patients admitted to an ICU develop delirium, which increases the mortality risk (Salluh et al., 2015). Patients with COVID-19 are at increased risk of delirium due to multiple factors, including (1) direct central nervous system (CNS) invasion, (2) induction of CNS inflammatory mediators, (3) secondary effect of noncerebral organ system failures, (4) effect of sedative strategies, (5) prolonged mechanical ventilation, (6) immobilization, and (7) isolation without family (Kotfis et al., 2020). Accordingly, a high rate of delirium (up to 84%) has been reported in critically ill patients with COVID-19 infection (Helms et al., 2020a). Basic research suggests a link between the circadian clock and delirium. In animal models, constant light exposure, inflammation, and midazolam exposure can induce delirium-like phenotype (i.e., impaired executive and memory function) and reduced expression of PER2 in the SCN. This delirium-like phenotype was abolished by the PER2 enhancer nobiletin (Gile et al., 2018).

Although it is clear that the circadian clock is challenged in the ICU, the origin of circadian manifestations in critically ill patients cannot be definitively attributed to the ICU environments. For example, severe illnesses may have direct effects on the circadian clock, regardless of the ICU environments. Furthermore, we have been operating with a bias that an intact circadian system is always optimal. This is partly due to observations of disruptions of metabolism with short-term clock disruption and increased chronic pathologies with long-term disruption (Schernhammer et al., 2001; Scheer et al., 2009). Stress-induced disruption of circadian rhythms or behavioral quiescence may rather represent a distinct adaptive mechanism. If this is correct, then restoration of daily rhythms in physiologic variables may be harmful. In other words, the “optimal state of biological rhythms” during critical illness may be different from that in health.

### Incorporation of the Circadian Clock Into Critical Care Paradigms for Health and Knowledge

#### Discovering the State of the Circadian Clock in the ICU

Chronobiologists are often biased to think that the presence of a circadian rhythm is the preferred state. Here, we have identified an ambiguous situation with a testable hypothesis. Our alternative hypothesis is that supporting the circadian clock with zeitgebers will lead to entrainment and daily rhythms, and this in turn will lead to a better outcome. The null hypothesis would be that the presence of rhythms is an exacerbation and that the suppression of rhythms sometimes seen in serious illness is adaptive. These hypotheses could be tested descriptively via collection of continuous data from ICU stations and analysis for rhythmicity and appropriate entrained phase as correlated with outcome. The data routinely collected in the ICU—e.g., temperature, oxygen saturation, and heart rate—show circadian rhythms in normal individuals. It represents a huge amount of information if it can be harnessed and analyzed. The hypotheses could also be tested experimentally, for example, by examining whether the circadian phase, amplitude, or robustness in temperature rhythms is associated with patient outcomes from severe COVID-19.

Given that the mediators of the most common complications in COVID-19 (hypercoagulation/bleeding, cytokine storm) are extensively regulated by the circadian clock, it may be expected that they occur at discrete times of day. Understanding this would allow for preventive treatments. Without deeper knowledge of the endogenous circadian clock characteristics specifically in the ICU, correct determination of clock regulation of these complications is not possible. What is needed is a comprehensive atlas of potential circadian outputs in ICU patients that include minimally invasive measurements such as blood pressure, heart rate, body temperature, as well as serial peripheral blood sampling over at least two cycles. From this dataset, ICU-appropriate biomarkers of the circadian clock can be derived to determine the circadian profile of the patient (rhythmic/nonrhythmic, amplitude, phase) and to assess the internal time of greatest risk for complications and that of optimal medication dosing schedules.

#### Delivering a Zeitgeber Cycle in the ICU

It can be challenging to administer a high-amplitude zeitgeber, whether light/dark or administration of corticosteroids with a <24-h half-life, to sedated or septic patients. Glucocorticoid administration should be weighed against its potentially life-threatening side effects including immunosuppression, and creating a dark environment for the patient during the night may interfere with patient care. On the other hand, modifying timing of nutritional support is feasible in the ICU for many patients and could potentially entrain peripheral clocks. We know of no efforts to administer food in the ICU intermittently, mimicking normal eating behaviors, in sedated patients. How is feeding accomplished in the ICU and what does nutrition therapy look like? Clinical trials and guidelines on nutrition therapy during and after critical illness have largely focused on optimal timing (early vs late initiation), route of delivery (gastric vs jejunal vs parenteral), and caloric/protein target. Enteral nutrition (EN) is the preferred route of artificial nutrition therapy in critically ill patients. Initiation of nutrition support therapy in the form of early EN within 24-48 h is recommended in the critically ill patient who is unable to maintain volitional intake, unless there are reasons to delay EN such as enteral obstruction, active gastrointestinal bleeding, and compromised splanchnic circulation (Taylor et al., 2016; Singer et al., 2019).

Several methods of EN administration exist, including continuous, cyclic, intermittent, and bolus techniques. Continuous feeding involves hourly administration of EN over 24 h assisted by a feeding pump; cyclic feeding involves administration of EN over a time period of <24 h generally assisted by a feeding pump; intermittent feeding involves administration of EN over 20-60 min every 4-6 h via pump assist or gravity assist; and bolus feeding involves administration of EN over a 4- to 10-min period using a syringe or gravity drip.

In practice, pump-assisted continuous feeding is generally acceptable for critically ill patients to reduce EN-related complications such as aspiration, feeding intolerance/high gastric residual, underfeeding, and diarrhea. However, a limited number of studies have been conducted to support this practice (Hiebert et al., 1981; Kocan and Hickisch, 1986; Ciocon et al., 1992; MacLeod et al., 2007). Small randomized controlled studies comparing bolus to continuous feeding in ventilated critically ill adults have shown greater volume with fewer interruptions in continuous feeding but no significant difference between feeding techniques with regard to patient outcome (Hiebert et al., 1981; Kocan and Hickisch, 1986; Ciocon et al., 1992; Bonten et al., 1996; Steevens et al., 2002; MacLeod et al., 2007). Based on existing evidence, the Society of Critical Care Medicine (SCCM) and American Society for Parenteral and Enteral Nutrition (ASPEN) suggest switching from bolus to intermittent EN in cases of feeding intolerance, whereas European Society for Clinical Nutrition and Metabolism (ESPEN) recommends continuous rather than bolus EN (Grade B—strong consensus; Taylor et al., 2016).

Time-restricted feeding (TRF) is an approach to resetting biological rhythmicity via metabolic entrainment of peripheral tissues. It may be time to explore the possibility of pursuing circadian realignment by optimizing the timing of feeding in relation to day-night cycle for critically ill patients receiving EN (Sunderram et al., 2014). Currently, no study has been performed to investigate the effects of timing of feeding in either bolus or continuous feeding in the ICUs. Future investigation will need to be equipped with a thoughtful selection of potential biomarkers of rhythmicity in peripheral tissues that can be collected in the clinical setting (e.g., microbiome, metabolomics/transcriptomics) and of clinically meaningful outcome variables.

## Summary

Taken together there are numerous aspects of the COVID-19 pandemic that may have a relationship to circadian and other biological rhythms, ranging from behavior of asymptotic carriers, organ physiology in the sick, the viral life cycle within infected cells, and the host immune response. Although biologically plausible, there has been limited attention to the potential protective effects of healthy sleep and circadian rhythms. Interestingly, the consideration of the available data on the ICU environment and critically ill patients points out the lack of data demonstrating if and how sleep and a synchronized clock contribute to healing. There are numerous examples of circadian disruption leading to illness but few examples of synchronization leading to healing. With judicious collection of time-stamped data, the current pandemic can be used to better understand how the circadian clock is involved in critical illness. We also propose that knowledge of the state of the circadian clock may be used to mitigate all stages of COVID-19.
